# Associations between smoking status and involvement of personal and professional relations among individuals reporting symptoms related to a diagnosis of lung cancer: a population-based study

**DOI:** 10.1186/s12889-022-14719-z

**Published:** 2022-12-06

**Authors:** Frederik Vognsgaard, Lisa Maria Sele Sætre, Sanne Rasmussen, Dorte Ejg Jarbøl

**Affiliations:** Research Unit of General Practice, Department of Public Health, University of Southern Denmark, Odense, Denmark

**Keywords:** Lung cancer symptoms, Primary health care, Healthcare-seeking, Relations, Smoking

## Abstract

**Background:**

Smoking is the leading cause of lung cancer, but individuals who currently smoke seek healthcare less frequently. This study of individuals reporting symptoms related to diagnosis of lung cancer has the following aims: 1) to explore the involvement of personal and professional relations; 2) to analyse whether age, sex and smoking status are associated with involving personal and professional relations; and 3) to analyse whether involving a personal relation is associated with healthcare-seeking.

**Methods:**

Data was extracted from a Danish population-based survey from 2012 with 100,000 randomly selected invitees 20 years or older. We describe the involvement of personal and professional relations among individuals experiencing four predefined symptoms indicative of lung cancer: prolonged coughing, prolonged hoarseness, shortness of breath and haemoptysis, either alone or in combination. Using multivariate logistic regression, we analyse the associations between involving personal or professional relations and various covariates (sex, age, smoking status). Moreover, we analyse the association between involving a personal relation and healthcare-seeking.

**Results:**

A total of 35,958 individuals over 40 years old completed the questionnaire. Of these, 5,869 individuals reported at least one lung cancer symptom. A higher percentage of participants with prolonged hoarseness and prolonged coughing reported no involvement of personal and professional relations (27.6% and 22.7%, respectively) compared to shortness of breath (12.4%). The most involved personal and professional relations were the spouse (46.2–62.5%) and the general practitioner (GP) (31.3–54.5%), respectively. Women and individuals in the oldest age group had higher odds of involving personal and professional relations. Individuals who currently smoke involved all relations less frequently than individuals who formerly,- and never smoked. Odds of contacting the GP or another doctor were three to seven-fold higher when a personal relation was also involved.

**Conclusion:**

Women and the oldest age group had higher odds of involving relations, whereas individuals who currently smoked tended to be less likely to contact any personal or professional relations. Involving a personal relation was associated with higher odds of healthcare-seeking. The findings could be useful for GPs in terms of identifying patients at risk of postponing relevant healthcare-seeking with potential lung cancer symptoms.

## Introduction

Lung cancer is common worldwide. Survival rates are highly dependent on stage of the disease at the time of diagnosis and timely diagnosis is crucial [1]. Focusing on optimizing the diagnostic process for patients presenting with symptoms, that might be related to lung cancer is therefore important [2]. Referral guidelines and fast-track investigation systems have been implemented in several countries to ensure that general practitioners (GPs) refer patients with alarming signs and symptoms for further investigation. Fast-track investigation only works, however, if individuals contact their GP when experiencing symptoms. Reducing the time between when symptom(s) are first noticed and when they are discussed with a GP is therefore essential to improve timely diagnosis [3]. Sociologists have suggested that healthcare-seeking behaviour is related to social life and social networks [4]. One study found that inclusion of social relations can contribute to the decision on whether or not to contact the GP [5]. Studies based on the Danish Symptom Cohort (DaSC) have previously reported that the involvement of personal relations is associated with increased likelihood of contacting a GP regarding urinary tract and gynaecological symptoms [6, 7]. This raises one of the hypotheses of this paper: involvement of a personal relation is associated with a higher likelihood of involving a professional relation.

The majority of patients with lung cancer are individuals with a current or former smoking history and the population of individuals who smoke worldwide is increasing [8]. Contrary in Denmark the percentage of individuals currently smoking have decreased during the last decade [9]. Sele et al. concluded that smoking is associated with less frequent contact to a GP among individuals reporting potential lung cancer symptoms [10]. If individuals who currently smoke contact GPs less frequently, they may also involve other relations less frequently. This leads to another hypothesis of this paper: individuals who currently smoke with symptoms related to lung cancer diagnosis may be less likely to involve both personal and professional relations, compared to individuals who never smoked.

This study of individuals reporting symptoms, related to diagnosis of lung cancerhas the following aims: 1) to explore the involvement of personal and professional relations; 2) to analyse whether age, sex and smoking status are associated with involving personal and professional relations; and 3) to analyse whether involving a personal relation is associated with healthcare-seeking.

## Methods

### Study design and population

This paper is based on data from the DaSC, a national, web-based, cross-sectional survey from 2012 [11]. In total, 100,000 Danish adults 20 years or older were invited to participate, randomly selected from the Danish Civil Registration System (CRS). All Danish citizens are registered in the CRS; the system provides a unique personal identification number along with information about date of birth, gender, place of residence, and other data [12].

Invitees received a letter explaining the purpose of the study and a code for secure web access to the questionnaire. Non-responders were reminded two weeks later by letter and again by telephone after an additional two weeks. Invitees were also offered the opportunity to complete the questionnaire by telephone with trained interviewers. The development, structure and testing of the questionnaire is meticulously described by Rasmussen et al. [11].

### Questionnaire

The objective of the DaSC was to investigate the prevalence of 44 symptoms and healthcare-seeking behaviour related to those symptoms in the general population. The respondents were asked whether they had experienced one or more of the predefined symptoms within the preceding four weeks; the question was phrased as: *“Have you experienced any of the following bodily sensations, symptoms, or discomforts within the past four weeks?”* Respondents were also asked when the symptom occurred for the first time (less than 1 month ago, 1–3 months ago, 3–6 months ago, or more than 6 months ago).

The present paper examines data related to the four specific symptoms as mentioned in the Danish lung cancer referral guideline: prolonged coughing, prolonged hoarseness, shortness of breath and haemoptysis [13, 14]. According to Danish lung cancer referral guidelines, coughing and hoarseness lasting longer than 4–6 weeks and 3–4 weeks, respectively, is regarded as prolonged [13]. To comply with these definitions, respondent who experienced coughing and hoarseness for the first time less than one month ago were excluded from further analyses. Non-specific symptoms such as tiredness and weight loss which may also be signs of lung cancer were not included in the present study.

Respondents were also asked whether they had talked to a relation about the reported symptom. Relations were classified in two categories: personal and professional. The question regarding personal relations was phrased: *“Which of the following personal relations have you talked to about the symptoms or discomforts? (Spouse/partner, children, parents, colleague/classmate, friend, neighbour, none and/or other).”* More than one answer could be selected.

Two questions defined professional relations: “*Have you contacted your GP about the symptoms or discomforts? (In person, by phone or by e-mail),”* and “*Which of the following other healthcare professionals have you talked to regarding the symptoms or discomforts? (Another doctor (practicing specialist, out-of-hours physician or hospital physician), physiotherapist/chiropractor, home help/district nurse, pharmacy staff, alternative therapist, none and/or other).”* More than one relation could be selected.

To determine availability of social network, we asked the following four questions: (1) ‘How often are you in contact with friends, acquaintances, or family that you do not live with? Contact indicate that you are together, talking with each other on the phone, writing to each other, etc.’ (daily or almost daily, once or twice a week, once or several times a month, less than once a month, never or I don’t know); (2) ‘If you become ill and need help with practical things, can you count on help from others? (Others means people you do not live with)’ (yes definitely, yes, maybe or no); (3) ‘Does it ever happen that you are alone, even if you want to be in the company of others?’ (yes often, yes once in a while, yes but rarely, no, never or almost never); (4) ‘Do you have someone to talk to if you have problems or need support?’ (yes often, yes once in a while, yes but rarely, no, never or almost never). Respondents were categorised as having an available social network if they answered often in contact with others (daily or almost daily, once or twice a week, or once or several times a month), having people available who can help (yes definitely or yes maybe), not being alone when desiring to be with others (never or almost never, rarely or once in a while) or having a person to talk to in case of problems (often, mostly or sometimes). The same definitions have been used elsewhere [6, 15].

Smoking status was ascertained in the question: *“Do you smoke? (Yes, every day; yes, at least once a week; yes, less than once a week; no, I have stopped; or no, I have never smoked).”* Smoking status was afterwards categorized as individuals who never, formerly,- or currently smoked [16, 17], the latter category including respondents currently smoking at any frequency.

### Statistical analyses

Only individuals 40 years or older were included in the analyses, in accordance with both national and international referral guidelines for lung cancer [13, 14].

For each potential lung cancer symptom, we used descriptive statistics to examine the involvement of personal and professional relations. Each symptom was analysed separately and stratified by smoking status. Per Danish legislation, reporting of data on individuals numbering fewer than five is not permitted, thus haemoptysis is only reported in some of the descriptive analyses due to few observations.

For each symptom, we analysed the associations between involving personal and professional relations and sex, age group, and smoking status, using multivariate logistic regression models. Moreover, the associations between involving a personal relation and contacting the GP or another doctor were analysed. To examine a possible interaction between overall social network and smoking status, we conducted sensitivity analyses, where we included an interaction term between smoking status and overall social network in the regression model for each outcome.

Professional relations were subdivided into: “the GP,” “another doctor,“ and “other professional relations” (physiotherapist/chiropractor, home carer/nurse, pharmacy staff, alternative therapist and other). The personal relations were subdivided into “spouse,” “family relations” (parents and children) and “non-family relations” (friend, colleague, neighbour and other). In all analyses the covariates were categorized as follows: age group (40–54 years, 55–69 years and ≥ 70 years), smoking status (never, former, and current smoking) number of symptoms reported (1–4 symptoms) and availability of social network. All statistical tests used a confidence interval of 95%. Analyses were performed using Stata statistical software version 17.

## Results

**Fig. 1:**
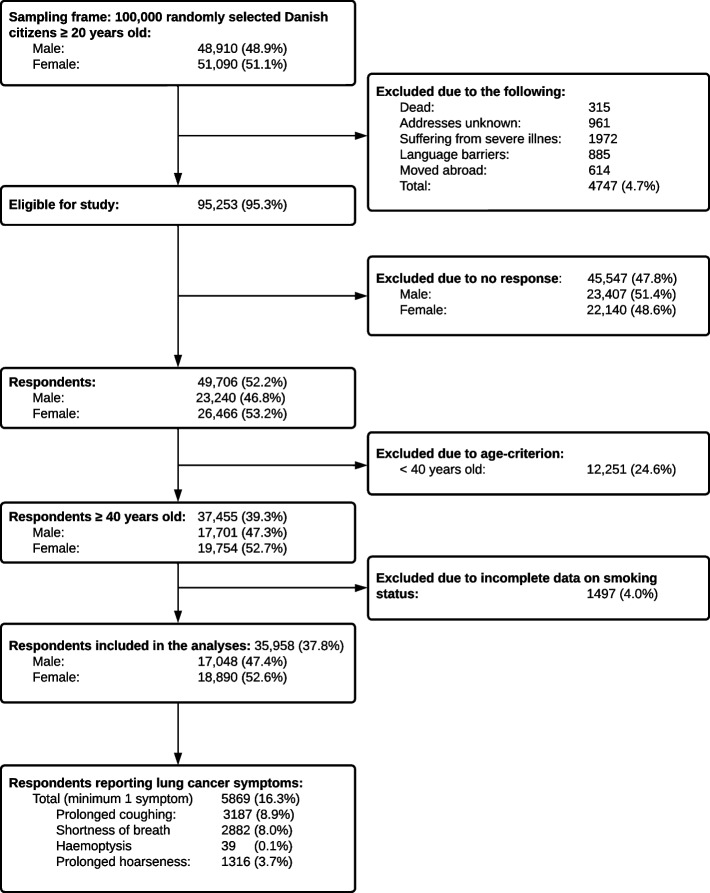
Flowchart

Of the 100,000 individuals invited to the DaSC, 4.7% were ineligible and excluded from the DaSC study population, Fig. 1. Of the 95,253 eligible, 49,706 (52.2%) Danes completed the DaSC survey. By only including respondents 40 years or older, a total of 37,455 respondents were included in the present study. Due to missing values for smoking status, the sample was further reduced to 35,958. A total of 5,869 had experienced at least one potential lung cancer symptom and were therefore eligible for further analyses, Fig. 1.

The prevalence of the various symptoms was 8.9% for prolonged coughing, 8.0% for shortness of breath, 0.1% for haemoptysis and 3.7% for prolonged hoarseness.

The most frequently involved personal relation was the spouse; involvement by symptom ranged from 46.2% (haemoptysis) to 62.5% (shortness of breath). Individuals not involving a personal relation, by symptom, ranged from 19% (shortness of breath) to 34% (prolonged hoarseness). The most frequently involved professional relation by symptom was the GP, ranging from 31.3% (prolonged hoarseness) to 54.5% (shortness of breath). Between 33.3% (haemoptysis) and 56.1% (prolonged hoarseness) of the individuals did not report involvement of any professional relations. 12.4% (shortness of breath) to 27.6% (prolonged hoarseness) of the individuals reported involvement of no personal or professional relations at all. See Table 1 for details.

**Table 1:** Characteristics of the study population and proportions of involvement of relations, classified by symptom

	**Study sample**	**Prolonged coughing**	S**hortness of breath**	**Haemoptysis**	**Prolonged hoarseness**
Total; n(%)	35,958(100.0)	3187(8.9)	2882(8.0)	39(0.1)	1316(3.7)
**Sex**					
Male	17,058(47.4)	1583(49.7)	1439(49.9)	24(61.5)	651(49.5)
Female	18,900(52.6)	1604(50.3)	1443(50.1)	15(38.5)	665(50.5)
**Age**					
40–54 years	14,567(40.5)	924(29.0)	996(34.6)	15(38.5)	327(24.8)
55–69 years	15,479(43.0)	1546(48.5)	1209(42.0)	15(38.5)	601(45.7)
70- years	5912(16.4)	717(22.5)	677(23.5)	9(23.1)	388(29.5)
**Smoking status**					
Never	15,063(41.9)	930(29.2)	824(28.6)	15(38.5)	439(33.4)
Former	13,389(37.2)	989(31.0)	1219(42.3)	16(41.0)	568(43.2)
Current	7506(20.9)	1268(39.8)	839(29.1)	8(20.5)	309(23.5)
**Number of symptoms**					
1	-	2021(63.4)	1870(64.9)	15(38.5)	625(47.5)
2	-	952(29.9)	799(27.7)	14(35.9)	483(36.7)
3	-	211(6.6)	210(7.3)	7(17.9)	205(15.6)
4	-	0	0	0	0
Available social network					
Yes	4040 (11.2)	476 (14.9)	510 (17.7)	11 (28.2)	210 (16.0)
No	31,918 (88.8)	2711 (85.1)	2372 (82.3)	28 (71.8)	1106 (84.0)
**Personal relations**					
Spouse	-	1851(58.1)	1802(62.5)	18(46.2)	675(51.3)
Child	-	669(21.0)	872(30.3)	11(28.2)	294(22.3)
Parent	-	133(4.2)	212(7.4)	< 5^*^	46(3.5)
Colleague/Classmate	-	259(8.1)	303(10.5)	< 5^*^	107(8.1)
Friend	-	461(14.5)	652(22.6)	8(20.5)	197(15.0)
Neighbour	-	119(3.7)	169(5.9)	0^*^	49(3.7)
Other personal	-	107(3.4)	160(5.6)	5(12.8)	46(3.5)
No personal	-	937(29.4)	549(19.0)	11(28.2)	447(34.0)
**Professional relations**					
General practitioner	-	1192(37.4)	1570(54.5)	21(53.8)	412(31.3)
Another doctor	-	750(23.5)	1176(40.8)	20(51.3)	339(25.8)
Physiotherapist/Chiropractic	-	34(1.1)	115(4.0)	< 5^*^	14(1.1)
Home carer/Nurse	-	29(0.9)	51(1.8)	< 5^*^	14(1.1)
Pharmacy staff	-	117(3.7)	79(2.7)	0	26(2.0)
Alternative therapist	-	67(2.1)	102(3.5)	0	21(1.6)
Other professional	-	193(6.1)	232(8.0)	< 5^*^	73(5.5)
No professional	-	1631(51.2)	898(31.2)	13(33.3)	738(56.1)
**No relations involved**					
None	-	725(22.7)	356(12.4)	7(17.9)	363(27.6)

^*^In accordance with Statistics Denmark, absolute numbers below 5 cannot be published

For all symptoms, current smokers involved fewest relations, compared with former and never smokers, as shown in Table 2.

**Table 2:** Relations involved by respondents experiencing lung cancer symptom, stratified by smoking status

**Smoking status**	**Prolonged coughing (*n* = 3187)**			**Shortness of breath (*n* = 2882)**			**Prolonged hoarseness (*n* = 1316)**		
	Never	Former	Current	Never	Former	Current	Never	Former	Current
Total; n(%)	932(29.0)	1000(31.1)	1285(39.9)	824(28.4)	1232(42.5)	843(29.1)	439(33.2)	572(43.2)	312(23.6)
**Personal relations**									
Spouse	616(66.1)	612(61.2)	639(49.7)	552(67.0)	832(67.5)	431(51.1)	256(58.3)	314(54.9)	108(34.6)
Family relations	262(28.1)	259(25.9)	233(18.1)	294(35.7)	443(36.0)	247(29.3)	114(26.0)	152(26.6)	54(17.3)
Non-family relations	220(23.6)	227(22.7)	261(20.3)	239(29.0)	420(34.1)	270(32.0)	92(21.0)	134(23.4)	72(23.1)
None	210(22.5)	261(26.1)	475(37.0)	143(17.4)	191(15.5)	216(25.6)	132(30.1)	165(28.8)	152(48.7)
**Professional relations**									
General practitioner	387(41.5)	449(44.9)	366(28.5)	431(52.3)	749(60.8)	401(47.6)	146(33.3)	190(33.2)	78(25.0)
Another doctor	254(27.3)	310(31.0)	196(15.3)	319(38.7)	599(48.6)	268(31.8)	123(28.0)	163(28.5)	54(17.3)
Other professional relations	137(14.7)	157(15.7)	115(8.9)	160(19.4)	233(18.9)	108(12.8)	45(10.3)	58(10.1)	27(8.7)
None	419(45.0)	415(41.5)	810(63.0)	264(32.0)	302(24.5)	335(39.7)	234(53.3)	301(52.6)	207(66.3)
**No relations involved**									
None	168(18.0)	182(18.2)	381(29.6)	102(12.4)	113(9.2)	142(16.8)	107(24.4)	126(22.0)	132(42.3)

### Involvement of personal relations

Women had statistically significant lower odds of involving a spouse for all symptoms compared with men. By contrast, women had statistically significant higher odds of involving both family relations and non-family relations, as shown in Table 3.

**Table 3:** Associations between sex, age, smoking status, and involvement of a personal relation and involvement of general practitioner, another doctor, spouse, family relations and non-family relations for prolonged coughing, shortness of breath and prolonged hoarseness

Prolonged coughing (*n* = 3187)										
	**Involvement of spouse**		**Involvement of family relations**		**Involvement of non-family relations**		**Involvement of GP**		**Involvement of another doctor**	
	Crude OR	Adjusted OR^a^	Crude OR	Adjusted OR^a^	Crude OR	Adjusted OR^a^	Crude OR	Adjusted OR^b^	Crude OR	Adjusted OR^b^
Sex										
Male (ref.)	1	1	1	1	1	1	1	1	1	1
Female	**0.70** **(0.61–0.81)**	**0.67** **(0.58–0.77)**	**2.16** **(1.82–2.56)**	**2.13** **(1.80–2.54)**	**2.53** **(2.12–3.02)**	**2.53** **(2.11–3.02)**	**1.26** **(1.10–1.46)**	**1.26** **(1.08–1.47)**	**1.20** **(1.02–1.41)**	1.18 (0.99–1.41)
Age										
40–54 years (ref.)	1	1	1	1	1	1	1	1	1	1
55–69 years	**1.35** **(1.14–1.59)**	**1.32** **(1.11–1.56)**	1.13 (0.93–1.38)	1.12 (0.91–1.37)	0.86 (0.71–1.04)	0.84 (0.69–1.03)	**1.42** **(1.19–1.69)**	**1.26** **(1.05–1.52)**	1.21 (0.99–1.47)	1.01 (0.82–1.25)
70- years	1.03 (0.85–1.25)	0.93 (0.75–1.14)	**1.52** **(1.21–1.91)**	**1.36** **(1.07–1.72)**	**0.61** **(0.47–0.77)**	**0.53** **(0.41–0.69)**	**2.14** **(1.75–2.62)**	**1.70** **(1.35–2.09)**	**1.36** **(1.08–1.71)**	0.92 (0.71–1.18)
Status of smoking										
Never (ref.)	1	1	1	1	1	1	1	1	1	1
Former	**0.81** **(0.67–0.97)**	**0.77** **(0.63–0.93)**	0.89 (0.73–1.09)	0.92 (0.75–1.13)	0.95 (0.77–1.17)	1.12 (0.90–1.40)	1.15 (0.96–1.37)	1.12 (0.92–1.36)	1.20 (0.98–1.46)	**1.27** **(1.03–1.57)**
Current	**0.51** **(0.43–0.60)**	**0.47** **(0.39–0.56)**	**0.57** **(0.46–0.69)**	**0.63** **(0.51–0.77)**	0.85 (0.67–1.01)	0.87 (0.71–1.08)	**0.56** **(0.47–0.67)**	**0.68** **(0.56–0.82)**	**0.48** **(0.39–0.59)**	**0.56** **(0.45–0.70)**
**Shortness of breath (*n* = 2882)**										
Sex										
Male (ref.)	1	1	1	1	1	1	1	1	1	1
Female	**0.55** **(0.47–0.64)**	**0.52** **(0.45–0.61)**	**1.79** **(1.54–2.10)**	**1.85** **(1.57–2.17)**	**2.08** **(1.77–2.44)**	**2.18** **(1.86–2.57)**	0.91 (0.79–1.06)	0.98 (0.84–1.14)	**0.81** **(0.70–0.94)**	0.87 (0.74–1.02)
Age										
40–54 years (ref.)	1	1	1	1	1	1	1	1	1	1
55–69 years	1.18 (0.99–1.40)	1.13 (0.94–1.36)	**1.67** **(1.39–2.02)**	**1.73** **(1.43–2.09)**	0.96 (0.81–1.15)	0.96 (0.80–1.16)	**2.06** **(1.74–2.44)**	**1.82** **(1.52–2.18)**	**1.75** **(1.47–2.09)**	**1.49** **(1.24–1.80)**
70- years	**0.75** **(0.61–0.91)**	**0.66** **(0.53–0.81)**	**2.30** **(1.87–2.83)**	**2.31** **(1.86–2.88)**	**0.70** **(0.57–0.87)**	**0.67** **(0.54–0.84)**	**3.10** **(2.53–3.80)**	**2.73** **(2.20–3.39)**	**2.15** **(1.76–2.62)**	**1.75** **(1.41–2.18)**
Status of smoking										
Never (ref.)	1	1	1	1	1	1	1	1	1	1
Former	1.02 (0.85–1.24)	0.97 (0.79–1.18)	1.01 (0.84–1.22)	0.96 (0.79–1.17)	**1.27** **(1.05–1.53)**	**1.51** **(1.23–1.84)**	**1.41** **(1.18–1.69)**	1.20 (0.99–1.45)	**1.50** **(1.25–1.79)**	**1.35** **(1.11–1.63)**
Current	**0.52** **(0.42–0.63)**	**0.46** **(0.37–0.57)**	**0.75** **(0.61–0.92)**	**0.79** **(0.64–0.97)**	1.15 (0.94–1.42)	1.25 (1.01–1.55)	0.83 (0.68–1.00)	0.89 (0.72–1.09)	**0.74** **(0.60–0.90)**	**0.79** **(0.63–0.97)**
**Prolonged hoarseness (*n* = 1316)**										
Sex										
Male (ref.)	1	1	1	1	1	1	1	1	1	1
Female	**0.68** **(0.55–0.85)**	**0.66** **(0.53–0.83)**	**2.27** **(1.75–2.95)**	**2.46** **(1.87–3.23)**	**2.78** **(2.11–3.67)**	**2.94** **(2.21–3.90)**	1.17 (0.92–1.47)	1.16 (0.90–1.49)	**1.32** **(1.03–1.69)**	**1.37** **(1.04–1.79)**
Age										
40–54 years (ref.)	1	1	1	1	1	1	1	1	1	1
55–69 years	**1.58** **(1.20–2.06)**	**1.43** **(1.08–1.90)**	1.23 (0.88–1.73)	1.28 (0.91–1.82)	0.89 (0.65–1.22)	0.97 (0.70–1.34)	1.29 (0.95–1.75)	1.16 (0.84–1.60)	1.36 (0.99–1.88)	1.21 (0.85–1.71)
70- years	1.32 (0.98–1.77)	1.10 (0.81–1.51)	**2.11** **(1.49–3.00)**	**2.09** **(1.44–3.02)**	**0.67** **(0.47–0.96)**	0.70 (0.48–1.02)	**1.90** **(1.37–2.62)**	**1.55** **(1.09–2.19)**	**1.58** **(1.12–2.23)**	1.22 (0.84–1.77)
Status of smoking										
Never (ref.)	1	1	1	1	1	1	1	1	1	1
Former	0.81 (0.62–1.05)	0.80 (0.61–1.03)	1.03 (0.78–1.37)	1.16 (0.86–1.56)	1.15 (0.85–1.56)	**1.53** **(1.11–2.10)**	1.00 (0.77–1.30)	0.95 (0.71–1.26)	1.02 (0.78–1.35)	1.06 (0.78–1.42)
Current	**0.38** **(0.28–0.51)**	**0.36** **(0.26–0.48)**	**0.60** **(0.42–0.86)**	**0.66** **(0.45–0.96)**	1.13 (0.80–1.60)	1.18 (0.82–1.70)	**0.67** **(0.48–0.93)**	0.81 (0.57–1.15)	**0.54** **(0.38–0.77)**	0.69 (0.47–1.01)

*OR* Odds Ratio, *GP* General Practitioner, *ref* reference

^a^Adjustments were made for possible confounders: sex, age groups, smoking status, and number of symptoms, and available social network

^b^Adjustments were made for possible confounders: sex, age groups, smoking status, involvement of a personal relation and number of symptoms

^*^Bold indicates significance level at 5%

The oldest age group had lower odds of involving a spouse regarding shortness of breath (Odds Ratio (OR) = 0.66, 95%CI [0.53–0.81]), and their non-family relations regarding all symptoms. Moreover, the oldest age group had statistically significant higher odds of involving family relations, for all symptoms.

The age group of 55–69 years had statistically significant higher odds of involving family relations when experiencing shortness of breath and a spouse when experiencing prolonged coughing and hoarseness.

Individuals who currently smoked had lower odds of involving a spouse, ranging from an OR of 0.36 (95%CI [0.26–0.48]) to an OR of 0.47 (95%CI [0.39–0.56]) for prolonged hoarseness and prolonged coughing, respectively. Further, individuals who currently smoked had lower odds of involving family relations for all symptoms (OR = 0.63, 95%CI [0.51–0.77] for prolonged coughing, OR = 0.79, 95%CI [0.64–0.97] for shortness of breath and OR = 0.66, 95%CI [0.45–0.96] for prolonged hoarseness). Individuals who formerly smoked showed the same tendency of lower odds of involving a spouse, which was statistically significant for prolonged coughing (OR = 0.77, 95%CI [0.63–0.93]). Contrary to the findings regarding individuals currently smoking, individuals who formerly smoked had higher odds of involving non-family relations for shortness of breath (OR = 1.51, 95%CI [1.23–1.84]) and for prolonged hoarseness (OR = 1.53, 95%CI [1.11–2.10]) compared with individuals who never smoked, as shown in Table 3.

### Involvement of professional relations

The odds of involving the GP were higher and statistically significant in the oldest age groups for all three symptoms. The same tendency was seen for the 55–69 age group, however, this finding was not statistically significant for prolonged hoarseness.

Individuals who currently smoked had lower odds of involving the GP (OR = 0.68, 95%CI [0.56–0.82]) for prolonged coughing compared with individuals who never smoked. The same tendency was found for individuals currently smoking involving another doctor, a finding that was statistically significant for prolonged coughing (OR = 0.56, 95%CI [0.45–0.70]) and shortness of breath (OR = 0.79, 95% CI [0.63–0.97]). For individuals who formerly smoked, the odds of involving another doctor were higher for prolonged coughing (OR = 1.27, 95%CI [1.03–1.57]) and shortness of breath (OR = 1.35, 95%CI [1.11–1.63]), as seen in Table 3.

Individuals involving a personal relation had higher odds of also having contacted the GP, ranging from an OR of 3.72 (95%CI [3.06–4.51]) for prolonged coughing to an OR of 4.16 (95%CI [3.05–5.67]) for prolonged hoarseness, as shown in Table 4. Similarly, the odds of contacting another doctor were higher when also involving a personal relation, with ORs of 6.45 (95%CI [4.85–8.56]), 6.29 (95%CI [4.679–8.25]) and 7.43 (95%CI [4.95–11.14]) for shortness of breath, prolonged coughing, and prolonged hoarseness, respectively, as shown in Table 4.

**Table 4:** Associations between involvement of the GP and another doctor and involvement of personal relations among individuals with lung cancer symptoms

	**Prolonged coughing (*n* = 3187)**				**Shortness of breath (*n* = 2882)**				**Prolonged hoarseness (*n* = 1316)**					
	**Involvement of GP**		**Involvement of another doctor**		**Involvement of GP**		**Involvement of another doctor**		**Involvement of GP**			**Involvement of another doctor**		
	Crude OR	Adjusted OR^a^	Crude OR	Adjusted OR^a^	Crude OR	Adjusted OR^a^	Crude OR	Adjusted OR^a^	Crude OR	Adjusted OR^a^		Crude OR		Adjusted OR^a^
**Involvement of a personal relation**														
No (ref.)	1	1	1	1	1	1	**1**	**1**	**1**	**1**	**1**		1	
Yes	**3.93 (3.26–4.74)**	**3.72 (3.06–4.51)**	**6.70 (5.07–8.85)**	**6.45 (4.85–8.56)**	**4.04 (3.29–4.95)**	**3.91 (3.17–4.83)**	**6.58 (5.05–8.60)**	**6.129 (4.79–8.25)**	**4.31 (3.19–5.83)**	**4.016(3.05–5.67)**	**8.00 (5.36–11.95)**		**7.43 (4.95–11.14)**	

*OR* Odds Ratio, *GP* General Practitioner, *ref* reference

^a^Adjustments were made for possible confounders: sex, age groups, smoking status, number of symptoms and available social network

^*^Bold indicates significance level at 5%

The sensitivity analyses showed no considerable interactions between smoking status and available social network regardless of the outcomes (data not shown).

## Discussion

Overall, the GP and the spouse were the most frequently involved professional and personal relations for individuals reporting potential lung cancer symptoms. Women and individuals in the oldest age groups had predominantly higher odds of involving personal and professional relations. Individuals who currently smoked tended to be less likely to contact any personal or professional relations compared with individuals who never smoked, however this finding was not statistically significant for all symptoms. Involving a personal relation was associated with three- to sevenfold increased odds of also involving the GP or another doctor.

## Strengths and limitations

One of the major strengths of this study is the large study sample of 100,000 individuals. The response rate of 52.2% was lower than in a similar Danish study [18], but higher than in two similar studies from the United Kingdom [19, 20].

The risk of misunderstanding and misinterpretation of survey questions was minimized through discussion of the construction of the questions with representatives from medical science, anthropology, and psychology, and through pilot and field testing the questionnaire [11].

Respondents were predominantly women and were slightly older compared with non-respondents, but the respondents were considered representative of the general Danish population [21]. The data used for this survey was collected in 2012, and we cannot rule out that the way individuals interact and share problems with their professional and personal relations has changed over the past decade. However, we still find it very important to report the data from our analysis of this large population-based study, as only sparse literature is available regarding symptom interpretation, healthcare-seeking behaviour and contacting personal relations. Moreover, it creates the possibility for future follow-up studies to explore changes in the observed behaviours over time.

To minimize selection bias, invitees were randomly selected through the CRS. Further, participants were offered the opportunity to complete the survey through a telephone interview as an alternative to the internet-based version; 3% of respondents chose this option.

The respondents were asked to recall symptom experiences within the previous 4 weeks and the accompanying involvement of any relations. The span of the recall period was selected since it was found reasonable to assume that people could adequately recall symptom experiences and involvement of relations within this timespan, thereby minimizing recall bias [22]. Nonetheless, recall bias is somewhat present in studies regarding self-reporting of previous events [23].

Connor Gorber et al. concluded in a systematic review that self-reported smoking is often underestimated [24, 25]. Self-reporting can be affected by stigmatization, shame, embarrassment, or other negative emotions [26–29]. A differential misclassification of the individuals who currently smoke could potentially cause an underestimation of the association between smoking and involvement of relations. These effects were minimized through the survey being primarily presented online, granting respondents a degree of anonymity. To decrease risk of stigmatising we have used first personal language when describing smoking status in the present study as suggested by e.g., Crocker and Smith [17].

The causality between symptoms and the involvement of personal and professional relations cannot be determined through a cross-sectional design, which does not allow accurate determination of the temporal order of variables. However, qualitative studies have suggested that family, friends, and social context can facilitate healthcare-seeking among individuals experiencing cancer symptoms [30–32].

Most individuals reporting symptoms potentially indicative of lung cancer will not have lung cancer. The symptoms will rather be self-limiting or caused by benign or chronic conditions [33]. However, investigating the many people with symptoms to diagnose the few with cancer is a prerequisite for the fast-track cancer pathways aiming to increase the chance of timely diagnosis. [34]. Thus, it is important to understand how potential lung cancer symptoms are interpreted and managed in the general population. It is unknown how often individuals who currently smoke or individuals who never smoked, respectively, should contact their GP when experiencing symptoms which might be indicative of lung cancer. However, it seems reasonable to assume that individuals who currently smoke, due to their increased risk of respiratory diseases, should contact their GP more often, and preferable not omit contacting the GP because they e.g., know or expect to have respiratory symptoms. This was emphasised in an update of the Danish lung cancer guideline in 2018, where a symptom described as ‘change in known cough’ was included [35]. The Danish lung cancer guidelines also include non-specific symptoms such as weight loss and tiredness as possible symptoms indicative of lung cancer. Exploring these symptoms are beyond the scope of this paper. Further, some individuals diagnosed with lung cancer do not present with any of the symptoms included in the lung cancer guideline. This group may potentially benefit from future screening programs currently being introduced in many countries [36, 37].

Denmark provides universal free health coverage, which warrants the assumption of a relatively high degree of involvement of professional relations in comparison to countries requiring self-payment [38]. This should be taken into consideration when comparing the findings of this paper to findings from other countries.

### Comparison to existing literature

A noticeable finding in this paper is that individuals reporting potential symptoms of lung cancer who involve a personal relation have higher odds of also involving the GP. This corresponds with previous studies on other symptoms, including cancer alarm symptom, which have suggested that advice from others increased the likelihood of seeking healthcare [5–7]. To the authors’ knowledge, no studies of such a large scale have previously examined the association between involving a personal relation and healthcare-seeking among people reporting potential lung cancer symptoms.

Another noticeable finding is that individuals who currently smoked had lower odds of involving nearly all relations, although this finding was not statistically significant for all symptoms. These findings are consistent with the findings of a population-based cohort from the United Kingdom, which indicated that current smoking is associated with less frequent social interactions with family and friends [39]. This may lead to the hypothesis that, current smokers have a less social network, than former and never smokers. Adjusting for available social network did however not change the results.

In the present study we choose to include both individuals who never-, formerly-, and currently smoked to enable comparison between groups, and to make investigation of low, but not no, risk groups possible. A recently published review by Os et al. emphasised the need of not only focusing on the high-risk group of individuals currently smoking, but also include and explore symptom appraisal and healthcare-seeking behaviour in regard to lung cancer symptoms among individuals who never smoked [40].

We found a difference in the likelihood of involving different relations for the various symptoms. This difference might be due to different impact or concern about the symptoms. Coughing might be a part of daily life for smokers, while shortness of breath is more intrusive, leading to higher impact or concern. It could also be associated with stigma, which is of particular significance for smoking and lung cancer [26–29]. Whether the symptoms induce different levels of concern or impact on daily life might be relevant in future research, to enhance the understanding of symptom-related factors affecting healthcare-seeking behaviour with symptoms, that might be indicative of lung cancer.

## Conclusion and implications

In this population-based study of individuals reporting symptoms related to diagnosis of lung cancer we showed that spouses and GPs were the most involved personal and professional relations. 

Women and individuals in the oldest age groups had higher odds of involving relations, whereas individuals who currently smoked tended to be less likely to contact any personal or professional relations. The likelihood of contacting a GP or another doctor was three- to seven-fold higher when a personal relation was also involved.

This study contributes new information regarding the association between involving a personal relation and healthcare-seeking for individuals reporting potential lung cancer symptoms. Individuals with no personal relationships, especially if currently smoking, may not contact healthcare professionals when experiencing lung cancer symptoms. This should be taken into consideration by GPs and other healthcare professionals during consultations or other types of contact. The findings could also be useful in public awareness campaigns aiming to provide information about the importance of timely diagnosis and therefore the urgency of healthcare-seeking for certain symptoms. Future studies could add to the understanding of the chronological order of involvement of relations and healthcare-seeking behaviour.

## Data Availability

The datasets generated and analysed in the current study are not publicly available due to the data protection regulations of the Danish Data Protection Agency, Statistics Denmark and the Danish Health and Medicines Authority. Access to data is strictly limited to the researchers who have obtained permission for data processing. This permission was granted to the Research Unit of General Practice, Department of Public Health, University of Southern Denmark. Further inquiries can be made to the PI Dorte Jarbøl email: DJarbol@health.sdu.dk.

## Abbreviations

GP: General practitioner
CRS: Civil Registration System
DaSC: Danish Symptoms Cohort
OR: Odds Ratio
